# PW01-031 – Treatment of FMF in middle and old age

**DOI:** 10.1186/1546-0096-11-S1-A84

**Published:** 2013-11-08

**Authors:** A Ayvazyan

**Affiliations:** 1 Yerevan State Medical University, Yerevan, Armenia

## Introduction

Current recommendations for the treatment of familial Mediterranean fever (FMF) are based largely on the observation of FMF patients receiving colchicine therapy in childhood and young age. The adequate colchicine therapy led to more and more patients survive to that age. In addition, there are national peculiarities of FMF. For example, in Armenia, even before the massive use of colchicine therapy, many patients survived to middle and old age.

## Objectives

We have investigated the corse of FMF in middle and old age, the incidence of miocardial infarction and the outcomes in case of myocardial infarction.

## Methods

Follow-up during 10-30 years.

## Results

Our research has shown that the risk of amyloidosis decreases with age, and the ability of colchicine to prevent attacks of FMF increases with age. Our research has also shown that in the absence of regular colchicine therapy increases the risk of myocardial infarction. In addition, myocardial infarction in patients with FMF is more severe, with a higher risk of death. With age the incidence of many diseases is increased, but their co-therapy with colchicine has not been studied.

## Conclusion

To date, may be recommended in middle and old age to take colchicine at a dosage that fully prevents the attacks of FMF in young age. If treatment is initiated in middle or old age, the dosage of colchicine should be higher than necessary to control the attacks of illness and indicators of inflammation. Untreated in young age FMF should be considered as a risk factor for CHD. Patients with FMF in the case of acute myocardial infarction should be observed over a long time and prevention of complications should be more intense. Urgently need to be initiated the multi-center and national studies on the combined treatment of FMF and the most common diseases in middle and old age.

## Disclosure of interest

None declared

